# Modeling *Bacillus cereus* Growth and Cereulide Formation in Cereal-, Dairy-, Meat-, Vegetable-Based Food and Culture Medium

**DOI:** 10.3389/fmicb.2021.639546

**Published:** 2021-02-17

**Authors:** Mariem Ellouze, Nathália Buss Da Silva, Katia Rouzeau-Szynalski, Laura Coisne, Frédérique Cantergiani, József Baranyi

**Affiliations:** ^1^Food Safety Microbiology, Food Safety Research Department, Institute of Food Safety and Analytical Sciences, Nestlé Research, Lausanne, Switzerland; ^2^Laboratory of Food Microbiology, Wageningen University & Research, Wageningen, Netherlands; ^3^Institute of Nutrition, University of Debrecen, Debrecen, Hungary

**Keywords:** *Bacillus cereus*, growth, cereulide formation, predictive microbiology, food, culture medium

## Abstract

This study describes the simultaneous *Bacillus cereus* growth and cereulide formation, in culture medium and cereal-, dairy-, meat-, and vegetable-based food matrices. First, bacterial growth experiments were carried out under a wide range of temperatures (from 9 to 45°C), using the emetic reference strain F4810/72, in the above-mentioned matrices. Then, the generated data were put in a modeling framework where the response variable was a vector of two components: the concentration of *B. cereus* and that of its toxin, cereulide. Both were considered time-, temperature- and matrix-dependent. The modeling was carried out in a series of steps: the parameters fitted in one step became the response variable of the following step. Using the square root link function, the maximum specific growth rate of the organism and the time to the appearance of quantifiable cereulide were modeled against temperature by cardinal parameters models (CPM), for each matrix. Finally, a validation study was carried out on an independent data set obtained in the same matrices and using various *Bacillus cereus* strains. Results showed that both growth and toxin-formation depended on the food matrix and on the environment but not in the same way. Thus, the matrix (culture medium), where the highest growth rate of *B. cereus* was observed, was not the medium where the shortest time to quantifiable cereulide occurred. While the cereal-based matrix generated the smallest growth rates (0.41-times smaller than culture medium did), quantifiable cereulide appeared in it at earlier times compared to the other tested matrices. In fact, three groups of matrices could be distinguished based on their ability to support cereulide formation (1) the cereal-based matrix (highest), (2) the culture medium and the dairy-based matrix (intermediate), and (3) the meat- and vegetable-based matrices (lowest). This ranking between the matrices is quite different from that based on their suitability to the growth of the organism. Our models can be used in HACCP studies, to improve shelf-life predictions and, generally, microbiological food safety assessments of products for which *B. cereus* is the main concern.

## Introduction

*Bacillus cereus* is a ubiquitous pathogen, commonly found in raw materials and occasionally in processed foods, such as rice, milk and dairy products, meat and meat products, pasteurized liquid eggs, ready-to-eat vegetables, and spices (Altayar and Sutherland, 2006; Wijnands et al., 2006; Hoton et al., 2009; Ceuppens et al., 2011; El-Arabi and Griffiths, 2013; Messelhäusser et al., 2014; Esteban-Cuesta et al., 2018; Fasolato et al., 2018; Park et al., 2018). *B. cereus* can produce two types of food poisoning illnesses: the diarrheal and the emetic syndromes appearing 8–16 h and 1–5 h after the ingestion of contaminated food, respectively (Messelhäusser and Ehling-Schulz, 2018). The diarrheal illness is induced by heat labile enterotoxins produced in the small intestine by vegetative cells (mainly through ingested spores germinating to become vegetative cells): the non-hemolytic enterotoxin (NHE), the hemolysin BL (HBL), as well as the cytotoxin K (CytK) (EFSA, 2016; Visiello et al., 2016). The emetic illness is triggered by the cereulide toxin, preformed in the food before ingestion (Ceuppens et al., 2011; Visiello et al., 2016).

Cereulide is thermostable as it withstands a heat treatment of 126°C for 90 min. It is not inactivated by the proteolytic enzymes of the gastrointestinal tract (not cleaved by pepsin or trypsin) and therefore can lead to toxigenesis (Agata et al., 1994, 1995; Rajkovic et al., 2008; Lücking et al., 2015; EFSA, 2016). It is important to avoid conditions leading to cereulide formation. Indeed, even if *B. cereus* cells were inactivated by appropriate treatments during food processing, cereulide would most likely remain intact and therefore present in the food at consumption.

Limited information is available on *B. cereus* strains and their potential to produce cereulide in food matrices as a function of different extrinsic and intrinsic parameters (e.g., temperature, pH, a_w_; see Rouzeau-Szynalski et al., 2020). For example, dairy- and cereal-based infant food formulae are known to promote cereulide synthesis due to their composition that is rich in vitamins and trace elements (Messelhäusser et al., 2014). It was also observed that cereulide production in culture medium is more likely to be observed in solid media than in liquid broth (Häggblom et al., 2002; Jääskeläinen et al., 2004). However, there is no clear guideline on the levels and combinations of factors to avoid cereulide formation. Instead, the food industry relies on *B. cereus* cell counts to set safety limits within their HACCP studies. In the absence of regulations linked to the cereulide toxin, the safety limit is generally set at 10^4^–10^5^ CFU/g or ml for *B. cereus* cell counts (EFSA, 2005, 2016).

However, Delbrassinne et al. (2011) mentioned that the correlation between *B. cereus* counts and cereulide concentration is not obvious. The type of food and the storage temperature seem to play a major role. However, there are not enough data for a systematic investigation of simultaneous *B. cereus* growth and cereulide formation in different matrices and at different incubation temperatures.

Modeling is a valuable tool to investigate this knowledge gap. Some *B. cereus* growth models were recently developed in specific matrices, for example in pasta and cooked rice (Juneja et al., 2019a,b), wet noodles (Ji-Young et al., 2011), or to predict *B. cereus* growth in reconstituted infant formulae (Buss da Silva et al., 2017; Bursova et al., 2018). But to the best of our knowledge, there are no published models to predict both *B. cereus* growth and cereulide formation simultaneously. The simultaneous description of the bacterial growth and toxin production raises new challenges not yet discussed in predictive food microbiology. Traditionally, the time-variation of bacterial response (e.g., its concentration) is described by a function termed the primary model. The effects of the environmental factors (e.g., temperature) on a parameter of the primary model is then described by a secondary model (Pérez-Rodríguez and Valero, 2013). In some cases, the metabolite formation rate is derived from the growth rate (Vereecken and Van Impe, 2002). In our case, however, the response variable of the primary model is, in fact, a vector consisting of two dynamic components: [*x(t), p(t)*]. The first is the time-dependent concentration of bacteria, the second is that of their toxic product. It is not straightforward to generalize standard predictive microbiology methods, developed for single response variables, to such vector-responses. For example, for a joint optimization, the components need to be transferred to the same scale. Our regression technique, using the logarithm link function, aims at minimizing the Root Mean Square of the relative differences between the primary model and the concentration data for growth and cereulide.

Baranyi et al. (2017) pointed out that a logical extension of the primary and secondary modeling should be the investigation how the parameters of the secondary model depend on the food matrix and/or the bacterial species, and this should be called tertiary modeling. In other words, the explanatory variables of tertiary modeling are typically category values. In fact, the very first tertiary models were published by Ross (1996), who assumed that the effect of the food matrix on the growth rate, compared to culture medium, can be characterized by a single scalar, a food-specific coefficient, called by the author the “bias factor” (that we call here the “correction factor” as it introduces just a slight modification to the model). Another commonly used experimentally proven tertiary model (see Pinon et al., 2004) is the assumption, utilized also by the present paper, that the minimum, maximum, and optimum temperatures for growth in the secondary model do not depend on the food matrix and are strain-specific.

In this paper, we describe the growth of the reference emetic *B. cereus* strain F4810/72 and its cereulide formation simultaneously, by vector-based predictive models, with special emphasis on the tertiary modeling of the effect of the matrix on the model parameters (including culture medium, cereal-, dairy-, meat-, and vegetable-based foods).

## Materials and Methods

### *Bacillus cereus* Growth

#### Strain Identification

An emetic *Bacillus cereus* strain isolated from an outbreak and previously described as a medium to high toxin producer (Carlin et al., 2006) was used in this study. This strain, identified as F4810/72 (or DSM4312) has been extensively characterized during recent years and is now referred to as one of the reference emetic strains (Ehling-Schulz et al., 2005; Carlin et al., 2013).

#### Inoculum Preparation

A stock culture was established using cryobeads stored at -20°C. For each experiment, one cryobead was sub-cultured in 10 ml Brain Heart Infusion broth (BHI, OxoidTM) and incubated for 8 h at 30°C. Then 100 μl of this culture was taken into another 9.9 ml of BHI broth and incubated for 18 h at 30°C. This overnight culture was then enumerated on Tryptone Soya Agar with Yeast extract (TSAYe) plates (OxoidTM) incubated for 24 ± 2 h at 30°C.

As a pre-trial, this experiment was repeated three times to assess the concentration obtained at the end of the incubation period. The average of the obtained values was used to assess the appropriate dilutions to prepare the inoculum. This procedure allowed to have more than 90% of vegetative cells.

#### Inoculation

Five matrices were studied to represent the possible contamination routes by *B. cereus*:

• (b) a sterile synthetic culture medium (BHI) to serve as reference, pH = 7.2, a_w_ = 0.997;
• (c) a cereal-based product (irradiated slurry composed of rice flour with water), pH = 6.3; a_w_ = 0.989;
• (d) a dairy-based product (reconstituted milk), pH = 6.8, a_w_ = 0.995;
• (m) a meat-based product (heat-treated meat emulsion), pH = 6.2, a_w_ = 0.985 and
• (v) a vegetable-based product (heat-treated vegetables puree), pH = 5.8, a_w_ = 0.997.

Solid food was portioned in 10 g portions while liquid matrices were distributed in 100 ml flasks.

The products were pre-warmed at the targeted temperatures before being spiked with the overnight inoculum to reach an initial target concentration of 10^2^ CFU/ml vegetative cells in each portion or flask.

At least three biological independent replicates obtained with three different inocula were performed for each matrix.

Non-inoculated samples were also prepared as negative controls for each product to check for the absence of natural contamination by *B. cereus* and absence of cereulide.

#### Growth Monitoring and Modeling

The growth of *B. cereus* vegetative cells was monitored in the different matrices at temperatures ranging from 9 to 45°C. Inoculated samples were sampled for viable counts on Bacara agar (Biomérieux), at appropriate sampling times, to describe the different phases of the growth curves.

For each matrix, each temperature and replicate, the growth curve was fitted by the Baranyi primary model (Baranyi and Roberts, 1994) using the DMFit MS Excel Add-in downloadable from www.combase.cc. When growth did not exceed 2 log increase, the growth was considered insignificant and no fitting was performed.

The secondary cardinal values model of Rosso et al. (1995) was chosen to describe the changes in the maximum specific growth rates (*μ*_*max*_, obtained from the bacterial growth curves), as a function of temperature. The square root transformation (SQRT) was used as a link function to stabilize the variance of the response variable along the temperature (Ratkowsky et al., 1983). The regression was conducted in R (R Core Team, 2019, version 3.6.1), using the nls Microbio package:


(1) $$\sqrt{\mu_{m\,a\,x}}=\sqrt{\mu_{o\,p\,t}.\gamma \,\left(T\right)}$$

where γ(*T*) is zero for *T*≤*T*_*min*_ and *T*≥*T*_*max*_, otherwise


(2) $$\begin{array}{ccc} \gamma \left(T\right) & = & \frac{\left(T-T_{\text{max}}\right){\left(T-T_{\text{min}}\right)}^{2}}{\left(T_{opt}-T_{\text{min}}\right)\left(\left(T_{opt}-T_{\text{min}}\right)\left(T-T_{opt}\right)-\left(T_{opt}-T_{\text{max}}\right)\left(T_{opt}+T_{\text{min}}-2T\right)\right)} \end{array}$$

The *T*_*min*_, *T_*o*__*pt*_*, and *T*_*max*_ parameters and *μ*_*opt*_ were estimated by fitting the data produced in BHI. For the sake of higher confidence in the parameter estimates of this BHI-based model, complementary experiments were performed using the OD-based binary dilution method of Cuppers and Smelt (1994) to obtain additional growth rate observations at temperatures 20, 28, 35, 40, 46.5, and 47.5°C in BHI. The *T*_*min*_, *T_*o*__*pt*_*, and *T*_*max*_ parameters were assumed to be independent of the matrix and fixed to the respective values obtained in BHI. Only the *μ*_*opt*_ parameter was made matrix-dependent, resulting in a series of *μ_*opt.f*_* parameters, where the index *f* runs through the *c, d, m, v* values (i.e., cereal, dairy-, meat-, and vegetable-based foods), while *μ*_*opt.b*_ denotes the estimate obtained in BHI.

A correction factor *C_*f*_* = *μ*_*opt*.*f*_/*μ*_*opt*.*b*_ (termed as bias factor by Ross, 1996) was calculated for each matrix to quantify the effect of the food on the optimum growth rate. This correction factor was assumed to be independent of the temperature. The correction factor compares the ability of a certain food matrix to support the growth of the studied strain in comparison to the growth obtained in culture medium. A correction factor close to 1 indicates similar growth of the studied strain in the food and in culture medium.

### Cereulide Quantification

#### Samples for Cereulide Analysis

At appropriate sampling times, samples were taken (1–2 ml for liquid matrices and or 1–5 g for solid matrices) and frozen for further cereulide analysis by liquid chromatography coupled with mass spectroscopy method (LC-MS/MS). The method was based on the ISO 18465 (2017) “Microbiology of the food chain- quantitative determination of emetic toxin (cereulide) using LC-MS/MS.” The method was modified to lower its Limit of Quantification (LoQ).

#### Cereulide Extraction

Samples were weighed in a 15 ml polypropylene tube (Falcon tubes, VWR International) to achieve a 1/10th dilution and then kept on ice. Cereulide was extracted by using acetonitrile (LC-MS grade, Merck) as described in the ISO 18465 (2017). An internal marked standard (^13^C_6_-cereulide) was added to reach a final concentration of 1.5 ng/ml.

The tube was then vigorously horizontally shaken at room temperature on an Orbital shaker (VXR basic Vibrax, VWR International) for circa 1 h at around 1,800 rpm (until adequate vortexing was achieved). After shaking, the tube was centrifuged (centrifuge Sigma-Aldrich) for 10 min at 1,000 G. The supernatant was filtered using a 0.22 μm Polytetrafluoroethylene (PTFE) syringe filter (PTFE filter, 0.22 μm, 13 mm minispike, VWR International) into a 2 ml amber glass vial (Agilent). The filtered extract was stored at -20°C until the injection.

#### Calibration Curve

The standard curve was prepared with synthetic cereulide (Chiralix) according to the ISO protocol (ISO 18465, 2017). A set of 11 levels ranging from 0.01 to 100 ng/ml of synthetic cereulide were prepared directly in 2 ml amber glass vials. The internal standard (^13^C_6_-Cereulide) was added to a final concentration of 1.5 ng/ml in acetonitrile to reach a final volume of 1 ml. The vials were stored at -20°C for 6 months to be used throughout the duration of the experiments.

#### Quantification by LC-MS/MS

The LC-MS/MS analysis was performed using an HPLC Agilent 1200 series coupled with Agilent QQQ 6460 (Agilent Technologies) according to the ISO protocol (ISO 18465, 2017). A column Supelco Discovery C18, 10 cm × 2.1 mm × 5 μm (Merck, 569220-U) was used with an isocratic elution of 10% of the mobile phase A (10 mM ammonium formate in water with 0.1% formic acid) and 90% of phase B (pure acetonitrile with 0.1% formic acid) and a flow rate of 0.4 ml/min at 40°C.

The mass spectrometer was operated in a positive ESI mode (ESI Low Tuning Mix G1969-85000, Agilent Technology) with a capillary voltage of 3,500 V, a nozzle voltage of 1,000 V, a gas temperature of 325°C, a gas flow of 10 L/min and a nebulizer at 35 psi. The sheath gas temperature and gas flow were at 250°C and at 8 L/min, respectively. MS/MS analysis was performed in multiple reaction monitoring (MRM) mode.

Cereulide peaks were confirmed when the relative deviation of q/Q area ratio of cereulide in the samples compared to the average ratio measured in standards did not exceed ±20%.

Agilent Masshunter Workstation Data Acquisition and Agilent QQQ quantitative Analysis (Quant-my-way) software were used for the acquisition and quantification, respectively.

The amounts of cereulide were obtained in ng/g of food sample. The limit of quantification (LoQ) was defined with spiked matrices based on a S/N ratio ≥ 6 for the cereulide qualifier. On the Agilent 6460, the LoQ was determined at 0.2 ng/g in the tested food matrices. The recovery rate was checked for all the runs to have at least 95% recovery. In addition, samples expected to have high or low cereulide levels, falling outside the calibration curve range, were diluted or concentrated, respectively.

### Cereulide Modeling

#### Defining and Scaling the Primary Model as a Vector

The primary model is a vector-function [*f_1_(t), f_2_(t)*], where the first component describes the time-variation of the *B. cereus* concentration, the second that of the cereulide. Following common standards in quantitative food microbiology, the natural logarithm of the *x(t)* bacterial concentrations were fitted by the *f*_1_ model of Baranyi and Roberts (1994). The standard error of such fit is a root-mean-square-average (RMSE) of the | ln *f_1_(t)*- ln *x(t)*| absolute differences, which is approximately the relative error between them:


RMSE|lnf(t)1-lnx(t)|≈Mean|f(t)1/x(t)-1|

This gives the idea that, for the *p(t)* cereulide measurements, too, their average relative differences from the model should be minimized:


Mean|f(t)2/c(t)-1|≈RMSE|lnf(t)2-lnp(t)|

For practical purposes, we used the logarithm transformation instead of the natural logarithm, but this did not make much difference, as only a scaling factor ln(10) ≈ 2.3 needed to be applied to some of the estimates. For example, for the bacterial curves measured in log cell concentrations, the fitted maximum rates needed to be multiplied by ln(10) to obtain the maximum specific growth rates, which is the highest slope of the curve fitted to the “ln(concentration) vs. time” data.

It is much less known about the true nature of the second component of the primary model than that of the first component. The measured cereulide concentrations are expected to be positively correlated with the bacterial concentration but, other than that, it is not easy to find a mechanistic model whose parameters, too, could be reliably estimated from measured data. Therefore, we took an empirical approach and only considered one of its parameters, the time to 0.2 ng cereulide/g of food. Accordingly, we called this the “time to quantifiable cereulide”, denoted by *t*_*cer*_ and the level of quantifiable cereulide was set to the mentioned LoQ value = 0.2 ng cereulide/g of food i.e., log *p_0_ ≈* −0.7 log (ng/g or ng/ml).

**FIGURE 1:**
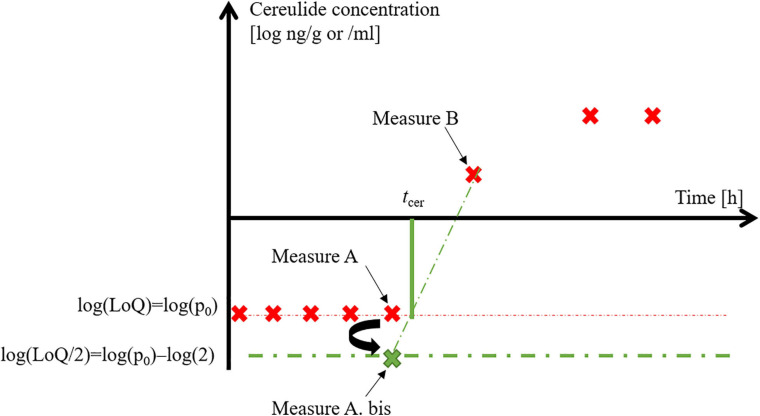
Linear interpolation to calculate t_cer_, the time to quantifiable cereulide level.


The *t*_*cer*_ parameter was estimated by the linear interpolation method of Hornung and Reed (1990), using the last observed point below the log *p*_0_ level and the first one above it (Point A and B in Figure 1). The log(*p*_0_/2) value was assigned to the last time point when the measured cereulide concentration was below the *p*_0_ level (a point called A.bis). Then the slope and intercept of the line combining the A.bis and B were used to estimate *t*_*cer*_:

(3) $$t_{c\,e\,r}=\frac{\log p_{0}-i\,n\,t\,e\,r\,c\,e\,p\,t}{s\,l\,o\,p\,e}$$

Just as the cardinal values model described the variation of the *μ_*max*_* parameter with the temperature, the *t*_*cer*_ parameter of the cereulide curves also depended on temperature, which is part of our investigations on the secondary model.

#### Secondary and Tertiary Modeling

The reciprocal of the “time to quantifiable cereulide level” is the same physical quantity as the maximum specific growth rate, and this gave the idea to use the “square root of the reciprocal” link function when regressing the *t*_*cer*_ estimates against temperature. The model can be written as


(4) $$\sqrt{1/t_{\mathrm{cer}}}=\sqrt{\frac{1}{t_{\mathrm{cer}.\mathrm{opt}}}\,\gamma_{\mathrm{cer}}\,\left(T\right)}$$

where γ(*T*) is zero for *T*≤*T*_*min*_ and *T*≥*T*_*max*_, otherwise


(5) $$\begin{array}{ccc} \gamma_{cer}\left(T\right) & = & \frac{\left(T-T_{\text{max}.cer}\right){\left(T-T_{\text{min}.cer}\right)}^{2}}{\left(T_{opt.cer}-T_{\text{min}.cer}\right)\left(\left(T_{opt.cer}-T_{\text{min}.cer}\right)\left(T-T_{opt.cer}\right)-\left(T_{opt.cer}-T_{\text{max}.cer}\right)\left(T_{opt.cer}+T_{\text{min}.cer}-2T\right)\right)} \end{array}$$

Here, the cardinal values of the cereulide secondary model (*T*_*min.cer*_, *T_*o*__*pt.cer*_*, *T*_*max.cer*_, and *t*_*cer.opt*_) are assumed to be matrix-specific, unlike in the case of the growth model.

However, we also investigated the restricted scenario when only the data generated at sub-optimum temperatures are considered, in which case the two-parameter- (*T_*min*_, b*) linear model of Ratkowsky et al. (1983) was used for fitting.

For tertiary modeling, the effect of the food matrix on the *b* parameter was quantified.

### Validation

The growth and cereulide models were validated against an additional set of data obtained with strains (Table 1) not used in the model development: B594 (medium cereulide producer, isolated from Chicken korma rice outbreak, also referenced as F3080B/87), B596 (low cereulide producer, isolated from cereals), B597 (Low cereulide producer isolated from meat), B626 (high cereulide producer, isolated from rice), B635 (a non cereulide producer control, also referenced as RIVM BC120). The validation was performed in the same food matrices, over the same range of temperatures used to develop the models (9–45°C).

**TABLE 1:** *B. cereus* strains used in the validation.

**Matrices**	**Growth**	**Cereulide**
BHI	B594**, B596*, B597*, B626***, B635^–^	B596* and B626***
Cereal-based	B626***	B626***
Dairy-based	B594**, B596*, B597*, B626***, B635^–^	B594**, B596*, B597*, B626***
Meat-based	B626***	B626***
Vegetable-based	B626***	B626***

**^–^**Not a cereulide producer, *low cereulide producer, **medium cereulide producer, ***high cereulide producer.

## Results

### *B. cereus* Growth and Cereulide Formation Kinetics

Growth was observed for all the temperatures between 12 and 45°C. At 9 and 10°C, growth was observed only for a selection of matrices (see Figure 3 for the growth rates obtained at all the tested temperatures).

**FIGURE 2:**
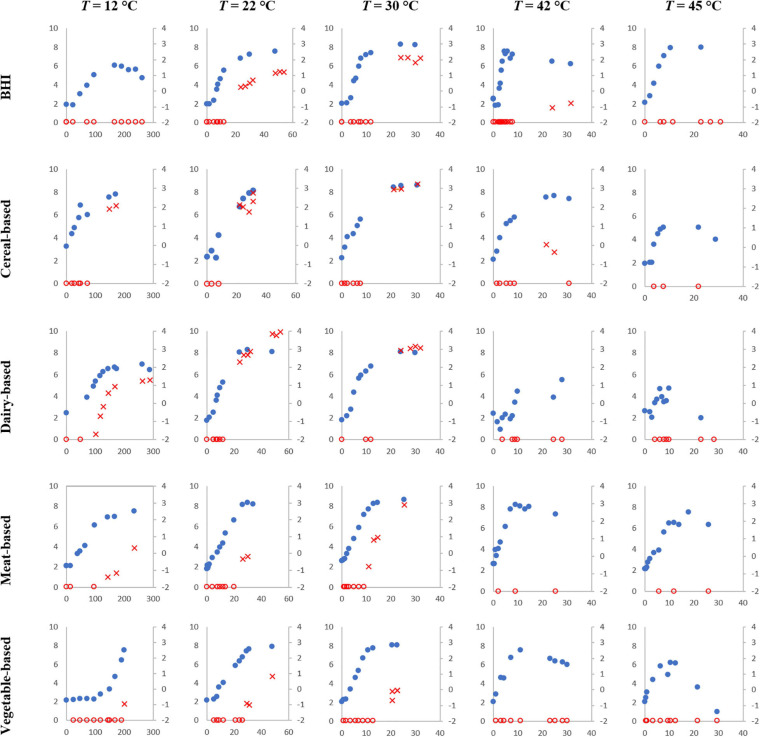
Simultaneous *B. cereus* (strain F4810/72) growth (blue dots, primary axis) in log CFU/g or /ml and cereulide formation (empty red circles when cereulide was lower than the Limit of Quantification LoQ and red crosses when cereulide was quantified, secondary axis) in log ng/g or /ml over time (h) at 12, 22, 30, 42, and 45°C in the tested matrices.

Figure 2 shows the growth and cereulide formation for selected temperature values (12, 22, 30, 42, and 45°C) for the five tested matrices, for one replicate (we call “replicate” an independent experiment prepared with a fresh inoculum from a given strain inoculated in a given matrix). As demonstrated, cereulide formation depends on both the temperature and the matrix.

At temperatures close to the growth boundaries (“happy growth” environment, from 22 to 37°C), cereulide formation was observed in all matrices, but the levels reached varied from matrix to matrix. For example, at 22°C, the maximum level for cereulide observed in the meat matrix was -0.16 log ng/g, while it was 0.35 log ng/g for the vegetable-based product, 1.21 log ng/ml for BHI, 2.76 log ng/g for the cereal-based matrix and 3.94 log ng/ml for the dairy matrix.

At temperatures that are far from the growth boundaries (outside of the “happy growth” environment), cereulide formation was observed only for some matrices. For example, at 12°C, no cereulide formation was observed in the vegetable-based product (maximum observation time 200 h) even though *B. cereus* populations reached 7.5 log CFU/g in this matrix. Cereulide formation was, however, observed in all the other matrices.

At 42°C, cereulide was not quantified up to 25 and 47 h in the meat-based and the vegetable-based products, respectively, while it was quantified at very low levels (close to LoQ) in the other matrices at different storage times.

Finally, at 45°C, growth was still observed in all matrices but, cereulide was not quantified in any, even well after the cells entered the stationary phase.

### Modeling *Bacillus cereus* Growth

**FIGURE 3:**
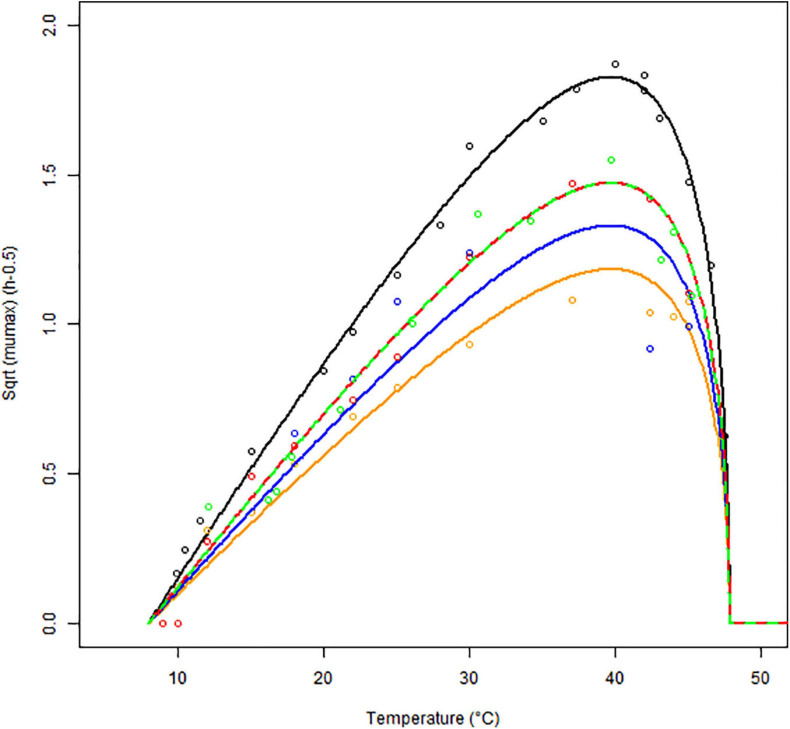
Secondary models (continuous lines) fitted to the maximum specific growth rates of *B. cereus* strain F4810/72 (empty symbols) as a function of temperature (°C) for BHI (black), cereal- (orange), dairy- (blue), meat- (red), and vegetable-based matrices (green). The Square Root (Sqrt) link function was used.

The growth rates obtained for each matrix at a given temperature were averaged and plotted against the respective temperatures. The results of the secondary model fitting are shown in Figure 3 and Tables 2, 3.

**TABLE 2:** Cardinal values of strain F4810/72 estimated on BHI data and assumed to be valid for food matrices, too.

***T*_*min*_** (°C)	**se(*T*_*min*_) (°C)**	***T_o__*pt*_* (°C)**	**se(*T_*opt*_*) (°C)**	***T*_*max*_ (°C)**	**se(*T*_*max*_) (°C)**	se(fit)* (h ^–^ **^0.5^)**
7.99	0.35	39.66	0.33	47.84	0.078	0.059

*In terms of sqrt (μ).

**TABLE 3:** Estimated μ_*opt.food*_ values and the derived correction factors for the tested matrices.

**Matrices**	***μ_*opt.f*_* (h^–1^)**	**se (*μ_*opt.f*_*) (h^–1^)**	***t*-value (−)**	se (fit)* (h^–1^)	***C*_*f*_ (−)**
BHI	3.33	0.076	43.8	0.059	1
Cereal-based	1.41	0.074	19.0	0.069	0.42
Dairy-based	1.78	0.269	6.61	0.180	0.53
Meat-based	2.16	0.084	25.7	0.058	0.65
Vegetable-based	2.17	0.110	19.7	0.091	0.65

*In terms of sqrt (μ).

In Figure 3, the square root (SQRT) of the growth rate follows the usual delta shape. It is close to linear between 15 and 25°C, starting from a *T*_*min*_ value, which is lower than the observed minimum temperature for growth. High non-linearity can be observed between 40 and 45°C, around the *T_*opt*_* temperature, where the curve reaches its maximum. Note that the growth rates obtained in food matrices at high temperatures, from plate count experiments, are usually associated with high uncertainty and can influence the secondary fit. Therefore, the cardinal values (*T*_*min*_, *T_*opt*_*, and *T*_*max*_) were estimated based on the BHI data only (Table 2) and for the other matrices, we assumed these cardinal temperature values to be conserved. This is a widely used tertiary-model-assumption (see Pinon et al., 2004).

The optimum growth rates *μ_*opt.f*_* were derived for each *f* matrix using the cardinal temperatures from Table 2. Table 3 shows that the highest optimum growth rate was reached in BHI, followed by the vegetable- and meat-based products (very similar estimates for their optimum growth rate), then the dairy products; the cereal-based products showing the lowest optimum growth rate.

Table 3 also shows the correction factors estimated for each food matrix based on the broth data. The meat and vegetable-based products have an identical correction factor (*C_*f*_* = 0.65) suggesting that *B. cereus* growth in these two matrices is comparable and higher compared to the dairy-based (*C_*f*_* = 0.53) or the cereal-based matrices (*C_*f*_* = 0.42).

Notice that the smallest standard error of fit associated to the *μ*_*opt.f*_ parameter was obtained for BHI. This is explained by the assumption that the food matrix does not affect the cardinal temperatures, therefore the fitting procedure was restricted to the estimation of the *μ*_*opt.f*_ parameter only while the other parameters *T*_*min*_, *T*_*o*__*pt*_, and *T*_*max*_ were fixed to the values obtained for BHI. However, in culture medium, all the four model parameters were estimated, resulting in a better standard error of fit.

The significance of the estimates was quantified by *t-*statistics. In each case, the respective probabilities were less than 2.10^–16^, robustly signifying the existence of an optimum specific growth rate value for each matrix.

**FIGURE 4:**
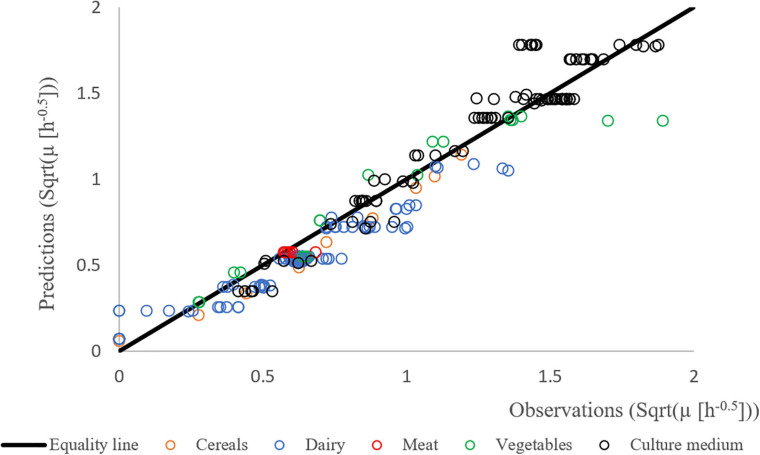
Predicted vs. observed growth rates of different strains of *B. cereus* in different matrices: BHI (black), cereal- (orange), dairy- (blue), meat- (red), and vegetable-based matrices (green). The Square Root (Sqrt) link function was used.

Figure 4 shows the results of the validation growth study with a good agreement between the observed and the predicted growth rates. The observed data were generated independently from the data used to build the model (obtained with strain F4810/72), involving different strains (see Table 1), suggesting that the newly developed growth models including the concept of the correction factor can be used to predict the growth rates in a variety of matrices.

### Modeling the Time to the Appearance of Quantifiable Cereulide

**FIGURE 5:**
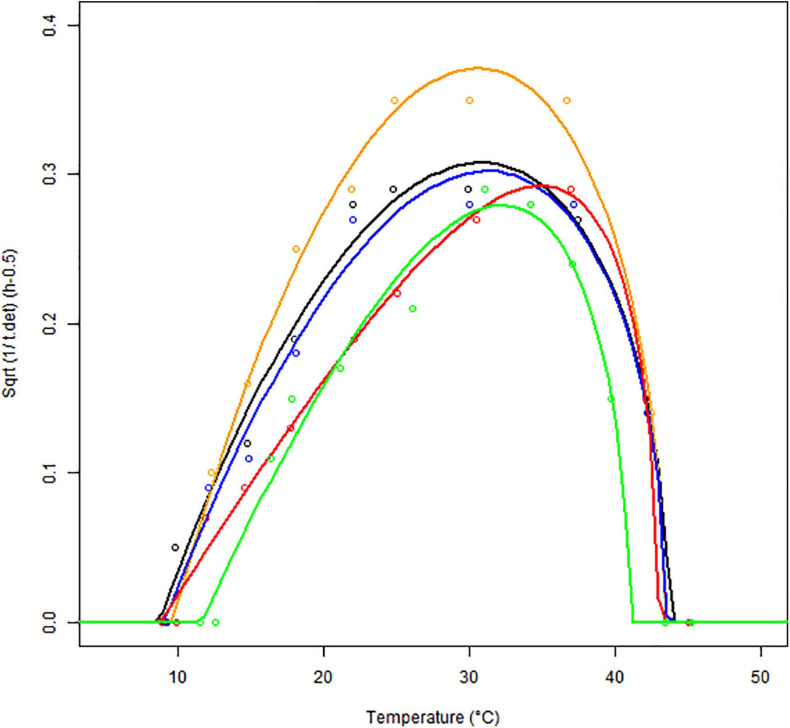
Fitting the square root (Sqrt) of the Cardinal Parameter Models (continuous lines) to square root of observed 1/t_cer_ values (empty symbols) as a function of temperature, in different matrices: BHI (black), cereal- (orange), dairy- (blue), meat- (red), and vegetable-based matrices (green) for strain F4810/72.

We used the reciprocal of the *t*_*cer*_ (time to the first appearance of quantifiable cereulide) as a response variable against the temperature. We generated the *t*_*cer*_ values as averages of three replicates, for each matrix, over the same temperature range used for bacterial growth (see Supplementary Material). The 1/*t*_*cer*_ values also followed a delta shape i.e., a convex-from-below function of temperature (see Figure 5, prepared with the square-root-scale). At low temperatures (9°C), no cereulide formation was detectable during the observation time of those experiments (17 days), so 1/*t*_*cer*_ = 0 was assigned to those temperatures. At sub-optimum conditions, 1/*t*_*cer*_ increased with the temperature to reach a matrix-specific maximum value at *T_*opt.cer*_*, then the curve dropped again, at super-optimum temperatures. The optimum temperature range for cereulide formation was between 22 and 37°C.

The parameter estimates are given in Table 4. They are in good agreement with the observations with similar goodness-of-fit indicators as for bacterial growth. The relative errors (standard error *per* parameter) for the 1/*t*_*cer.opt*_ estimates are lower than 10%, for all the five matrices and the standard errors of the cardinal temperature values for cereulide formation are all lower than 1°C.

**TABLE 4:** Secondary modeling results for cereulide formation by strain F4810/72 in different matrices.

**Matrices**	**1/*t*_*cer.opt*_ (h^–1^)**	**se (1/*t*_*cer.opt*_) (h^–1^)**	***RE* (**%**)**	***T*_*min.cer*_ (°C)**	**se (*T*_*min.cer*_) (°C)**	***T*_*o*__*pt.cer*_ (°C)**	**se (*T*_o__*pt.cer*_) (°C)**	***T*_*max.cer*_ (°C)**	**se (*T*_*max.cer*_) (°C)**
BHI	0.095	0.007	7	8.7	0.5	30.8	0.8	43.6	0.5
Cereal-based	0.138	0.007	5	9.6	0.4	30.5	0.5	43.3	0.2
Dairy-based	0.091	0.008	9	9.1	0.7	31.2	0.9	43.5	0.5
Meat-based	0.086	0.005	6	8.8	0.5	34.9	0.6	42.9	0.2
Vegetables-based	0.078	0.006	7	11.6	0.9	32.2	0.8	40.9	0.5
			Average	9.6	–	31.9	–	42.8	–
			Std.	1.2	–	1.8	–	1.1	–

Figure 5 and Table 4 show that the cereal-based matrix has the highest 1/*t*_*cer.opt*_ value. This means that, in optimum conditions, the time for *B. cereus* to produce cereulide to quantifiable levels is the fastest. It is estimated at 7 h in this matrix and at 13 h in the vegetable-based matrix. These results underline the importance of the matrix-effect, when assessing the time to cereulide formation.

Table 4 shows variability of the *T*_*min.cer*_ parameter. It varies from 8.7°C in BHI to 11.6°C in the vegetable-based matrix, suggesting that the minimum temperature for cereulide formation is also matrix-specific. The maximum temperature for cereulide formation *T*_*max.cer*_ also varies with the matrix from 40.9°C for the vegetables based to 43.6°C in the cereal-based products.

**FIGURE 6:**
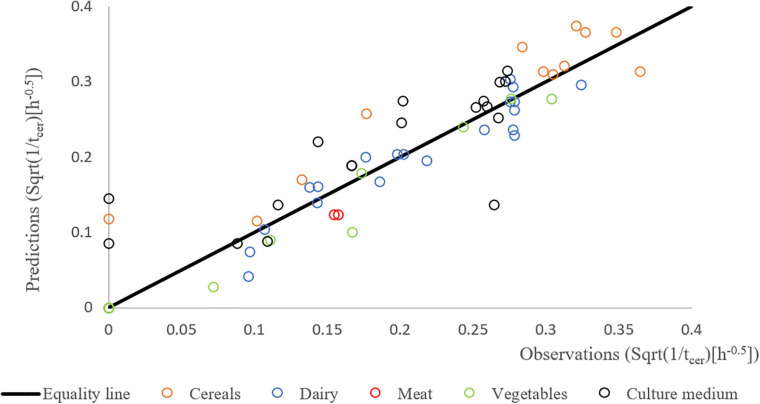
Predicted vs. observed square root (Sqrt) of 1/t_cer_ for the different strains of the validation study in the different matrices: BHI (black), cereal- (orange), dairy- (blue), meat- (red), and vegetable-based matrices (green).

Figure 6 shows good agreement between the predictions and the independent observations suggesting that the cereulide model is sufficiently robust when used to predict times to quantifiable cereulide formation in the studied matrices.

## Discussion

This work provides new models to describe *B. cereus* growth and time to cereulide formation by emetic strain F4810/72 when artificially inoculated in BHI, cereal-, dairy-, meat- and vegetable-based products.

The growth and cereulide formation were simultaneously monitored over time and appropriate primary models were fitted to the data generated in different matrices. The secondary modeling was based on the cardinal values approach both for the growth and cereulide models.

We also introduced a new concept here, inasmuch we considered the primary model as an [*f_1_(t)*, *f_2_(t)*] dynamic vector-function. The first component, *f_1_(t)*, described the concentration of bacteria, *x(t)*, while that of the cereulide, *p(t)*, was described by the second component *f_2_(t)*. We used the natural logarithm link function for both, to transform them to the same scale. This is closely related to the objective that our fitting should minimize the expected relative error between the measured concentrations and their primary models, for both cases. These models can be used to derive the maximum storage times before the concentration of *B. cereus* and cereulide reach certain threshold levels in the different tested matrices.

For the growth models, we estimated the cardinal temperature values for the studied strain, based on data generated by plate counts in BHI medium. Those cardinal values were within the confidence intervals reported by Carlin et al. (2013), for the same strain and medium. They were thus used as the cardinal parameters of the BHI model and for the other food matrices. We proposed a correction factor *C*_*f*_ to link the optimum growth rates of each matrix to the optimum growth rate in BHI. The validation study, conducted on independent data with other *B. cereus* strains, showed that the developed models are robust and can be used with confidence to predict growth rates in the different tested matrices. Based on the correction factors calculated in this study for the major food categories, it becomes possible to deduce from predicted growth rates obtained at given temperatures in broth, more realistic and matrix-specific growth rates to be used in future *B. cereus* risk assessments.

In the cereulide models, we described the variation of the 1/*t*_*cer*_ values with the temperature analogously to the above secondary model for the specific growth rate of *B. cereus*. We observed noticeable, matrix-specific differences between the cardinal values for *B. cereus* growth and cereulide production.

This observation is in agreement with other studies indicating that the temperature range for growth of emetic *B. cereus* strains is broader than the temperature range for cereulide formation (Carlin et al., 2006; Apetroaie-Constantin et al., 2008) and that cereulide production was low or undetectable at temperatures around 37°C but optimal between 15 and 30°C (Finlay et al., 2000). The results also showed that the “1/*t*_*cer*_ vs. temperature” curves were less skewed and exhibited wider plateaus around their optimum range (around 30°C) than the growth rate models did (around 40°C).

**FIGURE 7:**
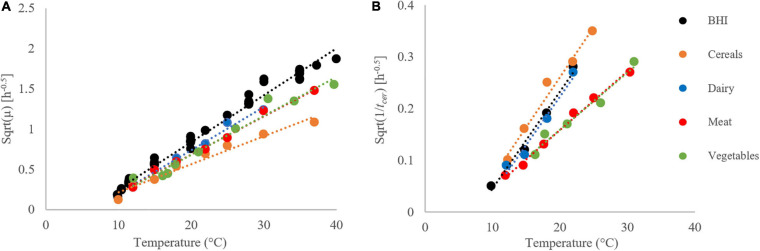
Growth rates **(A)** and time to first cereulide formation **(B)** for strain F4810/72 at the sub-optimum temperature range, fitted by the Ratkowsky model. The Square Root (Sqrt) link function was used.

As the measurements are much more reliable at the sub-optimum range of temperature, we separated the respective 1/*t*_*cer*_ values, and modeled them with the two-parameter Ratkowsky function (Ratkowsky et al., 1983), which is valid in that temperature range, and linear there: *b(T-T_*min*_)*. The results are shown by Figure 7. The goodness of fit of this simple model is remarkable, keeping in mind that the definition of *t*_*cer*_, is much more arbitrary and data-dependent, than that of the maximum specific growth rate for the bacteria. The role of this parameter is similar to the lag time for bacterial growth, which depends on the pre-inoculation history of the cells (Baranyi, 1994). This gives a hint that the *B. cereus* cells had the same gene-expression potential, before the inoculation, to produce cereulide, however, it is the substrate in the growth environment that determines, at what rate this potential is realized.

The figure also demonstrates that, at suboptimal conditions, the bacteria grow the fastest in BHI, while the cereulide formation is the fastest in the cereal-based matrix. This finding casts doubt whether the cereulide formation is strongly correlated with the growth of *B. cereus*, with consequences on food safety assessment, as the hazard is not its growth but is toxin production.

The *b*-parameters of the Ratkowsky model (see Table 5) can be used to quantify relationships between these identified secondary models; indeed, tertiary modeling is about the very analysis of such relationships. Within the growth models, the correction factor for an *f* food, for the suboptimal temperature range, is equivalent to *C*_*f*_′ ≈ (*b*_*f*_/*b*_*b*_)^2^ (see Baranyi et al., 2017). The difference between *C*_*f*_′ and *C*_*f*_ depends on how close the data trend is to linearity.

**TABLE 5:** Estimates of the *b*-parameters of Ratkowsky, for the growth and cereulide models.

**Matrices**	***b* (growth) [(1/√h)/°C]**	***b* (cereulide) [(1/√h)/°C]**
BHI	0.059	0.012
Cereal-based	0.038	0.015
Dairy-based	0.052	0.012
Meat-based	0.048	0.0090
Vegetable-based	0.048	0.0094

The *b*-values for the cereulide, too, can be used to characterize the relationships between the various matrices:

Group (1): “Cereal-based matrix,” with the highest cereulide formation rate and the lowest growth rate, this means that cereulide will be produced faster in this matrix compared to the others even though the growth rate would be lower;
Group (2): “Dairy and BHI” with intermediate cereulide formation rates and the highest growth rates; this means that cereulide in these two matrices will be produced more slowly compared to the cereal-based matrix but the growth rates will be the highest;
Group (3): “Meat and Vegetables,” with low cereulide formation rates and moderate growth rates; this means that cereulide is expected to be produced slowly in those matrices while the growth rates are higher than the cereal-based matrix and lower than the Dairy and BHI matrices.

Interestingly, meat and vegetables have the lowest cereulide formation rates. Literature shows that emetic strains tend to be abundant in vegetables but rare in meat products (Ehling-Schulz et al., 2015). We showed here that the toxin formation rate in this meat-based matrix is the lowest while the growth rate is not (see Table 5). This finding is also in alignment with Jääskeläinen et al. (2004) who reported that adding meat peptone to the growth medium of *B. cereus* increased its growth capabilities only, without no significant effect on cereulide formation.

Our study showed that cereulide formation was faster and reached higher levels in the cereal-based matrix compared to the other tested matrices. This means that when evaluating the exposure to the emetic toxin cereulide, considering *B. cereus* growth alone does not provide enough information to assess the safety of a given food product. To do so, it is important either to quantify the toxin directly or to be able to identify via prior studies the link between bacterial counts and the time to quantifiable cereulide levels in each food category.

This study has shown differences in cereulide formation in the tested matrices. It is out of the scope of our investigations, why we observe those differences. The literature provides guidance on potential factors supporting cereulide formation such as temperature, pH, oxygen, certain types of nutrients, sugars, fiber, even some micro-nutrients, food structure (liquid or solid) but without a direct indication of their mechanism of action, or a ranking of the most important ones.

For example, we know that cereulide formation requires oxygen (Finlay et al., 2000). For cereal-based foods like rice, Jääskeläinen et al. (2004) showed that storage under nitrogen atmosphere reduced the production of cereulide by a factor of 140 times and that the presence of L-Leucine and L-valine stimulated cereulide production by a factor of 10 or 20 in nutrient agar or rice water agar. But it is unclear how oxygen levels influence cereulide formation in presence of complex nutrient environments such as those encountered in food systems. Moreover, Dommel et al. (2011) reported that increasing salt levels in liquid medium lowers cereulide production without affecting the bacterial growth rate. Another study reported that long chain polyphosphates used as preservatives in the food industry are likely to negatively influence the *ces* gene transcription and therefore cereulide synthesis (Frenzel et al., 2011) but the role of other preservatives such as organic acids is yet to be investigated.

Finally, Jääskeläinen et al. (2004) investigated the effect of the structure (solid vs. liquid culture medium) on cereulide formation and showed that solid media support more cereulide production compared to liquid media. However, in our study the cereal-based matrix (viscous liquid) supported the cereulide formation more than solid food did (such as the meat-based or vegetable-based foods, indicating that the effector is not only the structure of the food, much rather a complex interaction between several factors.

One can think about performing meta-analysis to identify and rank those influencing factors. However, gathering information from the literature on cereulide production in different foods and under different conditions is challenging because the methodologies and the analytical methods are varied. Methodologies sometimes focus on the production of cereulide by a certain time and do not study the whole kinetic for cereulide formation. As the exact time for cereulide formation is often non-observable, it is difficult to compare the different studies. Moreover, some focus on cereulide quantification at a given bacterial concentration or at a specific phase of growth (generally stationary phase). However, we showed here, and it has also been reported in the literature, that the start of cereulide production is matrix-specific and not directly linked to the growth phases. In fact, in cereal-based matrices and some dairy matrices, cereulide was already quantified when *B. cereus* growth reached 4 log CFU/g or /ml while for the other matrices, cereulide was not detected before the limit of 5 log CFU/g or /ml. Besides, from an analytical perspective, it is not easy to compare the results obtained by the different cereulide methods (bore sperm motility assays, monkey feeding tests, Hep 2 cells, LC-MS/MS). The publication of the ISO standard (ISO 18465, 2017) will promote the harmonization efforts and will make it easier to compare the results obtained from future studies.

Ensuring a high level of safety for food products is the number one priority of the food companies. Our study proposed validated models to predict *B. cereus* growth and cereulide formation in the main food categories. However, it is important to have a better understanding of the underlying *ces* gene expression mechanism to form cereulide and the impact of the extracellular factors on this synthesis. This is key to provide safe-by-design foods using the appropriate combination of hurdles to prevent cereulide formation in foods and better manage the associated risks.

## Data Availability Statement

All datasets generated for this study are included in the article/Supplementary Material, further inquiries can be directed to the corresponding author/s.

## Author Contributions

ME: conception, execution, and writing up. NB and KR-S: execution and writing up. LC and FC: execution. JB: writing up. All authors contributed to the article and approved the submitted version.

## Conflict of Interest

ME, KR-S, LC, and FC were employed by company Nestlé. The remaining authors declare that the research was conducted in the absence of any commercial or financial relationships that could be construed as a potential conflict of interest.
