# Corrigendum: Modeling *Bacillus cereus* Growth and Cereulide Formation in Cereal-, Dairy-, Meat-, Vegetable-Based Food and Culture Medium

**DOI:** 10.3389/fmicb.2021.755736

**Published:** 2021-09-22

**Authors:** Mariem Ellouze, Nathália Buss Da Silva, Katia Rouzeau-Szynalski, Laura Coisne, Frédérique Cantergiani, József Baranyi

**Affiliations:** ^1^Food Safety Microbiology, Food Safety Research Department, Institute of Food Safety and Analytical Sciences, Nestlé Research, Lausanne, Switzerland; ^2^Laboratory of Food Microbiology, Wageningen University & Research, Wageningen, Netherlands; ^3^Institute of Nutrition, University of Debrecen, Debrecen, Hungary

**Keywords:** *Bacillus cereus*, growth, cereulide formation, predictive microbiology, food, culture medium

In the original article, there was an error. Equations 2 and 5 had a mistake in their denominator.

A correction has been made to **Materials and Methods**, Bacillus cereus Growth, Growth Monitoring and Modeling section to change Equation 2 to the following expression:


$$\begin{array}{l} \gamma (T)=\frac{\left(T-T_{\max}\right){\left(T-T_{\min}\right)}^{2}}{\left(T_{opt}-T_{\min}\right)(\left(T_{opt}-T_{\min}\right)\left(T-T_{opt}\right)}\\ \;-\left(T_{opt}-T_{\max}\right)\left(T_{opt}+T_{\min}-2T\right)) \end{array}$$

A correction has been made to **Materials and Methods**, Cereulide Modeling, Secondary and Tertiary Modeling section to change Equation 5 to the following expression:


$$\begin{array}{l} \gamma_{cer}(T)=\frac{(T-T_{\max.cer}){\left(T-T_{\min.cer}\right)}^{2}}{(T_{opt.cer}-T_{\min.cer})(\left(T_{opt.cer}-T_{\min.cer})(T-T_{opt.cer}\right)}\\ \;-(T_{opt.cer}-T_{\max.cer})(T_{opt.cer}+T_{\min.cer}-2T)) \end{array}$$

The authors apologize for this error and state that this does not change the scientific conclusions of the article in any way. The original article has been updated.

## Publisher's Note

All claims expressed in this article are solely those of the authors and do not necessarily represent those of their affiliated organizations, or those of the publisher, the editors and the reviewers. Any product that may be evaluated in this article, or claim that may be made by its manufacturer, is not guaranteed or endorsed by the publisher.

