# Supplementary material for: Modeling Bacillus cereus Growth and Cereulide Formation in Cereal-, Dairy-, Meat-, Vegetable-Based Food and Culture Medium
Source: Front Microbiol. 2021 Feb 17;12:639546. doi: 10.3389/fmicb.2021.639546 (PMC7925994; doi:10.3389/fmicb.2021.639546)
Supplement: Supplementary file 1 [file Table_1.DOCX]

Supplementary material: Changes in the observed square root of 1/t_cer_ values as a function of temperature, in the studied different matrices: BHI, cereal-, dairy-, meat- and vegetable-based matrices for emetic *B. cereus* strain F4810/72.

| Matrix | Temperature (°C) | Sqrt (1/t_cer_) (h^-0.5^) |
| --- | --- | --- |
| BHI | 9.15 | 0 |
| BHI | 9.84 | 0.05 |
| BHI | 12.12 | 0.09 |
| BHI | 14.78 | 0.12 |
| BHI | 18.03 | 0.19 |
| BHI | 22.01 | 0.28 |
| BHI | 24.73 | 0.29 |
| BHI | 29.92 | 0.29 |
| BHI | 37.4 | 0.27 |
| BHI | 42.3 | 0.14 |
| BHI | 45 | 0 |
| Cereals | 8.89 | 0 |
| Cereals | 9.9 | 0 |
| Cereals | 12.29 | 0.1 |
| Cereals | 14.75 | 0.16 |
| Cereals | 18.1 | 0.25 |
| Cereals | 21.94 | 0.29 |
| Cereals | 24.83 | 0.35 |
| Cereals | 30.01 | 0.35 |
| Cereals | 36.61 | 0.35 |
| Cereals | 42.41 | 0.14 |
| Cereals | 45 | 0 |
| Dairy | 9.01 | 0 |
| Dairy | 9.24 | 0 |
| Dairy | 12.06 | 0.09 |
| Dairy | 14.82 | 0.11 |
| Dairy | 18.1 | 0.18 |
| Dairy | 22 | 0.27 |
| Dairy | 30.01 | 0.28 |
| Dairy | 37.1 | 0.28 |
| Dairy | 42.16 | 0.14 |
| Dairy | 45.04 | 0 |
| Meat | 8.99 | 0 |
| Meat | 9.95 | 0 |
| Meat | 11.93 | 0.07 |
| Meat | 14.59 | 0.09 |
| Meat | 17.68 | 0.13 |
| Meat | 22.05 | 0.19 |
| Meat | 25.04 | 0.22 |
| Meat | 30.42 | 0.27 |
| Meat | 36.93 | 0.29 |
| Meat | 42.06 | 0.15 |
| Meat | 45.05 | 0 |
| Vegetables | 11.49 | 0 |
| Vegetables | 12.6 | 0 |
| Vegetables | 16.37 | 0.11 |
| Vegetables | 17.8 | 0.15 |
| Vegetables | 21.1 | 0.17 |
| Vegetables | 26.1 | 0.21 |
| Vegetables | 31.03 | 0.29 |
| Vegetables | 34.2 | 0.28 |
| Vegetables | 37 | 0.24 |
| Vegetables | 39.7 | 0.15 |
| Vegetables | 43.38 | 0 |
| Vegetables | 45.25 | 0 |
